# The Impact of Lymph Node Ratio for Children with Wilms Tumors: A National Cancer Database Analysis

**DOI:** 10.3390/cancers17193276

**Published:** 2025-10-09

**Authors:** Ioannis A. Ziogas, Andrii Khomiak, Kaitlin E. Olson, Dimitrios P. Moris, Alexandria J. Robbins, Jenny Stevens, Shannon N. Acker, Jonathan P. Roach, Kristine S. Corkum, Nicholas G. Cost

**Affiliations:** 1 Division of Pediatric Surgery, Department of Surgery, University of Colorado Anschutz Medical Campus, Children’s Hospital Colorado, Aurora, CO 80045, USA; 2 Surgical Oncology Program, Children’s Hospital Colorado, Aurora, CO 80045, USA; 3 Department of Pediatrics, University of Colorado Anschutz Medical Campus, Children’s Hospital Colorado, Aurora, CO 80045, USA; 4 Department of Surgery, MedStar Georgetown Transplant Institute, Washington, DC 20007, USA; 5 Division of Urology, Department of Surgery, University of Colorado Anschutz Medical Campus, Children’s Hospital Colorado, Aurora, CO 80045, USA

**Keywords:** Wilms tumor, nephroblastoma, lymph node, survival, pediatric cancer, lymph node ratio, nephrectomy

## Abstract

**Background**: Lymph node status is a prognostic factor in Wilms tumor, and adequate lymph node sampling is strongly recommended. This study investigates the impact of lymph node ratio (LNR) (number of positive to examined lymph nodes) on overall survival in children with resected Wilms tumors. **Methods**: This retrospective National Cancer Database analysis included children (<18 years) who underwent resection with lymph node sampling for unilateral, non-metastatic Wilms tumor. **Results**: Among 2206 patients, the median age was three years, the median tumor size was 10.5 cm, and the median number of examined nodes was five. A total of 82.1% of patients had an LNR of 0, 5.4% had an LNR < 0.2, and 12.5% had an LNR ≥ 0.2. In multivariable Cox regression, LNR ≥ 0.2 was associated with worse survival (HR = 1.75, 95%CI: 1.03–2.97, *p* = 0.04), along with increasing age (HR = 1.11, 95%CI: 1.05–1.17, *p* < 0.001) and tumor size (HR = 1.03, 95%CI: 1.00–1.06, *p* = 0.03). **Conclusions**: LNR is an independent prognostic factor in Wilms tumor and may refine risk stratification and guide treatment decisions.

## Simple Summary

Wilms tumor is the most common renal cancer in children, and survival has greatly improved with advances in surgery, chemotherapy, and radiation. However, some children still have poor outcomes, and better ways to predict survival are needed. One possible marker is the lymph node ratio, which measures how many cancerous lymph nodes are found compared to the total number of lymph nodes removed during surgery. In this study, we analyzed national data from over 2000 children with Wilms tumor to see if lymph node ratio could help predict survival. We found that children with a higher ratio had worse outcomes, especially when more than one in five sampled lymph nodes contained cancer. These results suggest that lymph node ratio could be used alongside current staging methods to more accurately assess risk and guide treatment decisions for children with Wilms tumor.

## 1. Introduction

Wilms tumor, or nephroblastoma, is the most common renal tumor and the fourth most common cancer in children [1,2]. Surgical resection, preferably with the intention to remove the entire tumor in one piece, is the mainstay of treatment for Wilms tumor [3,4,5]. The collaborative efforts of cooperative groups have led to significant improvements in survival for children with Wilms tumors through advances in multimodal treatment, including a combination of surgery, chemotherapy, and radiation [6,7,8,9]. However, there is still variability in outcomes for children with more advanced disease, highlighting the need for improved prognostic markers.

Initially reported in 1980, locoregional lymph node involvement is a well-known predictor of poor outcomes in children with Wilms tumors [10,11]. Traditional staging systems categorize nodal involvement as positive or negative, but recent studies suggest that more refined metrics, such as the lymph node ratio (LNR)—defined as the number of positive lymph nodes divided by the total number of examined lymph nodes—may provide additional prognostic value [12,13,14]. In other cancers, LNR has emerged as a significant predictor of survival, with higher ratios correlating with worse outcomes [15,16,17,18,19,20,21,22,23].

The aim of this study was to evaluate the prognostic significance of LNR in children with resected Wilms tumor and to determine whether it provides additional stratification beyond conventional nodal status positivity in the contemporary era. By analyzing overall survival in relation to LNR, we seek to refine risk assessment in Wilms tumor and potentially inform future treatment strategies.

## 2. Materials and Methods

### 2.1. Study Design and Setting

We conducted a retrospective cohort study including all children with Wilms tumor registered in the National Cancer Database (NCDB). The NCDB is a joint project of the Commission on Cancer of the American College of Surgeons and the American Cancer Society and incorporates about 70% of all newly diagnosed cancers in more than 1500 hospitals accredited by the Commission on Cancer in the USA [24]. The NCDB includes data on demographic, clinical, and pathological characteristics, data on tumor characteristics (histology, behavior, stage, sequence of malignancy, etc.), as well as treatment data, and overall survival. The NCDB has been widely used in the assessment of management and outcomes in the field of pediatric surgical oncology [25,26]. As this study represents non-human subjects research, the Institutional Review Board deemed it exempt from review.

### 2.2. Study Participants

Children (<18 years) diagnosed with Wilms tumor who underwent surgical resection and lymph node sampling were identified from the NCDB Pediatric Renal Participant User Files between 2004 and 2019 using the International Classification of Diseases for Oncology, 3rd Edition kidney site code “C64.9” and histology code “8960”. Patients were excluded if they had a previous malignancy, metastatic or bilateral disease, if they did not undergo surgical resection, or if data regarding the time from diagnosis to last patient contact, vital status at last patient contact, race, ethnicity, insurance, metastasis, tumor size, tumor laterality, or treatment were unavailable. LNR was defined as the number of positive lymph nodes divided by the total number of examined lymph nodes.

### 2.3. Statistical Analysis

Categorical variables were reported in frequencies and percentages, while between-group comparisons were performed with the chi-square test. Continuous variables were reported as median and interquartile range (IQR), while between-group comparisons were performed with the Kruskal–Wallis test.

The primary outcome of interest was overall survival, defined as the duration from the date of diagnosis until the date of last patient contact or death. The R package cutpointr (Supplemental File S1) was used to find an optimal cut-point for LNR that maximized the sum of sensitivity and specificity regarding overall survival using the area under the curve (AUC). The 1-, 3-, and 5-year overall survival rates were determined using the Kaplan–Meier method, and the log-rank test was used to assess differences in univariable analysis. Multivariable Cox regression modeling was implemented to adjust for a priori selected clinically important variables including LNR, age, sex, race/ethnicity, tumor size, and receipt of chemotherapy and radiation. Two Cox regression models were constructed: the first one included LNR as a categorical variable (0, <0.2, ≥0.2), and the second one included LNR as a continuous variable both with and without restricted cubic splines. Model comparison was conducted using the likelihood ratio test (LRT) and Akaike Information Criterion (AIC). Cohort development and statistical analyses were conducted using Stata IC 16.0 (StataCorp LLC, College Station, TX, USA) and R (version 4.4.2).

## 3. Results

### 3.1. Demographic and Clinical Characteristics

**Figure 1:**
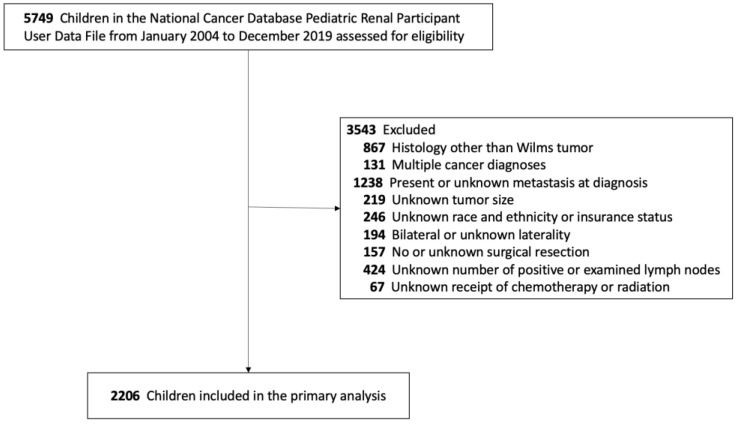
Cohort assembly flow diagram.

**Figure 2:**
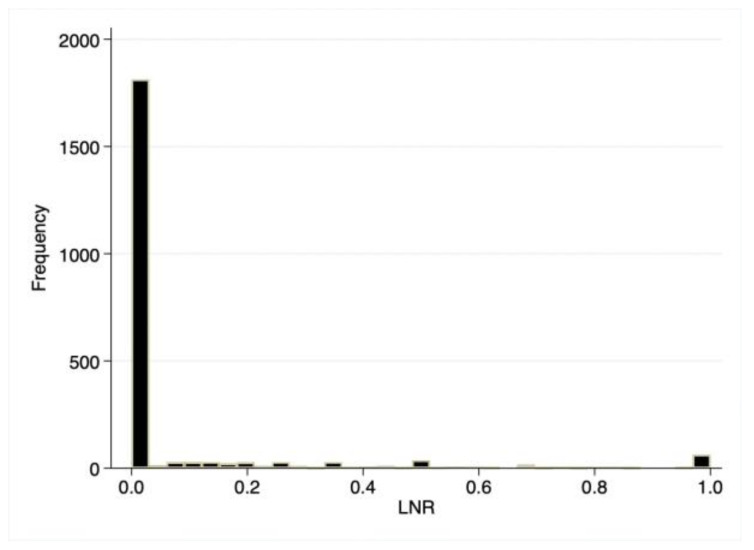
Histogram of lymph node ratio.

We evaluated a total of 2206 children with resected, unilateral, non-metastatic Wilms tumor (Figure 1). The median number of harvested lymph nodes for the entire cohort was five, and the median number of positive lymph nodes was zero. As a result, the median LNR was 0 (IQR: 0.0–0.0; Figure 2), with 1811 (82.1%) having an LNR of 0, 120 (5.4%) having an LNR < 0.2, and 275 (12.5%) having an LNR ≥ 0.2. The median age for the entire cohort was 3.0 years (IQR: 1.0–5.0), 51.6% were female, 61.4% were White, 55.4% had private insurance, and 95.6% had a Charlson–Deyo score of 0. However, patients with an LNR of 0 had smaller primary tumor size compared to patients with LNR < 0.2 and ≥0.2 (median 10.2 vs. 12.0 vs. 11.5 cm, *p* < 0.001).

The most common surgical procedure performed was radical nephrectomy with 74.3%, followed by total nephrectomy with 18.0%, partial nephrectomy with 4.0%, and nephrectomy with en bloc organ resection with 3.6%, without a statistically significant difference amongst groups (*p* = 0.28). A smaller proportion of patients with an LNR of 0 had positive surgical margins compared to patients with LNR < 0.2 and ≥0.2 (12.7% vs. 21.7% vs. 25.1%, *p* < 0.001). Although there was no statistically significant difference regarding receipt of chemotherapy amongst the three groups (89.7% vs. 95.0% vs. 92.7%, *p* = 0.06), a significantly smaller proportion of patients with an LNR of 0 received radiation compared to patients with LNR < 0.2 and ≥0.2 (39.2% vs. 96.7% vs. 92.4%, *p* < 0.001). Detailed patient and clinical data for the entire cohort and by LNR group are shown in Table 1.

### 3.2. Overall Survival

**Figure 3:**
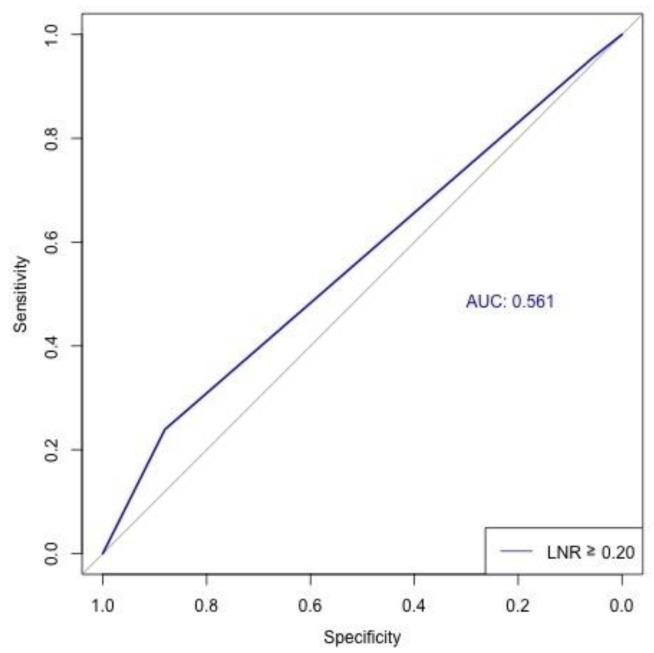
Area under the curve identifying lymph node ratio of 0.2 as the optimal cutoff.

**Figure 4:**
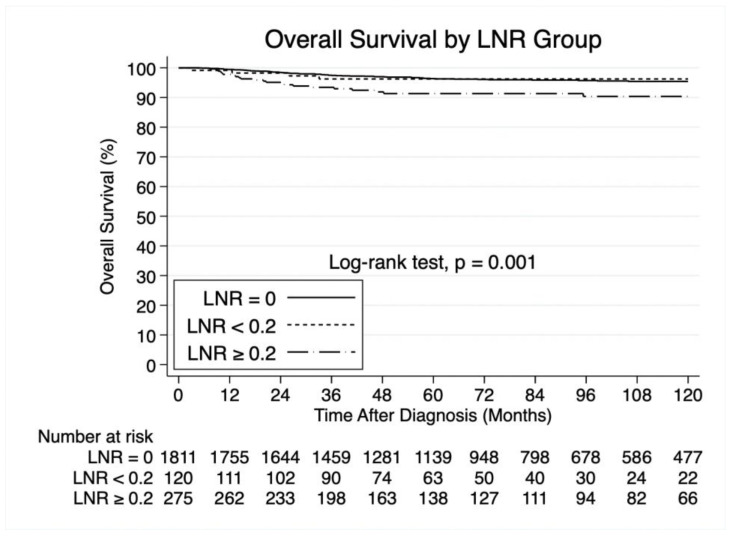
Kaplan–Meier curves demonstrating differences in overall survival by lymph node ratio group.

Based on AUC analysis (Figure 3), we identified that the optimal cutoff for LNR was 0.2, corresponding to a modest AUC value of 0.56. LNR ≥ 0.2 was associated with significantly inferior overall survival (log-rank test *p* = 0.001; Figure 4). The 1-, 3-, and 5-year overall survival rates were 99.4%, 97.5%, and 96.4% for LNR of 0; 99.1%, 96.2%, and 96.2% for LNR < 0.2; and 97.8%, 93.4%, and 91.3% for LNR ≥ 0.2.

In multivariable Cox regression analysis treating LNR as a categorical variable, LNR ≥ 0.2 compared to LNR of 0 (hazard ratio [HR] = 1.75, 95% confidence interval [95%CI]: 1.03–2.97, *p* = 0.04), increasing age (HR = 1.11, 95%CI: 1.05–1.17, *p* < 0.001), and increasing tumor size (HR = 1.03, 95%CI: 1.00–1.06, *p* = 0.03) were associated with an increased risk of patient mortality, when adjusting for sex, race/ethnicity, and receipt of chemotherapy and radiation (Table 2).

In multivariable Cox regression analysis treating LNR as a continuous variable with and without restricted cubic splines, the model without restricted cubic splines demonstrated better fit. Increasing LNR (HR = 2.60, 95%CI: 1.27–5.32, *p* = 0.01), increasing age (HR = 1.11, 95%CI: 1.05–1.18, *p* < 0.001), and increasing tumor size (HR = 1.03, 95%CI: 1.00–1.06, *p* = 0.03) were associated with an increased risk of patient mortality, when adjusting for sex, race/ethnicity, and receipt of chemotherapy and radiation (Table 3).

## 4. Discussion

The present study assessed the prognostic significance of LNR in a population-level cohort of children who underwent surgical resection for unilateral, non-metastatic Wilms tumor using U.S. cancer registry data in the contemporary era. Our findings indicate the utility of using LNR as a risk stratification tool to predict inferior overall survival with a higher LNR overall being associated with worse outcomes, especially if ≥0.2. Additionally, we found that older age at the time of diagnosis and increased tumor size were also associated with inferior overall survival in this patient population.

Although lymph node status is a well-established prognostic factor for children with Wilms tumor, until recently lymph node involvement was primarily classified in a binary fashion of positive and negative status [27,28]. Therefore, LNR, also commonly referred to as lymph node density, which accounts for both the number of positive lymph nodes and the extent of lymph node dissection, has been proposed as a more robust prognostic marker. Consistent with prior studies evaluating the utility of lymph node density in the prognostication of Wilms tumors [12,13,14], our findings reinforce the prognostic significance of lymph node disease burden and emphasize the need for improved lymph node evaluation. A key consideration in interpreting LNR is the adequacy of lymph node sampling. A growing body of evidence has demonstrated that an insufficient lymph node yield can lead to understaging, which can affect survival [11,29,30]. Another analysis of the NCDB evaluating lymph node yield adequacy in favorable-histology Wilms tumor reported that between six and ten lymph nodes should be examined to optimize staging accuracy [12]. However, in our cohort, the median number of lymph nodes examined was five, which may limit the accuracy of LNR as a prognostic indicator.

Despite that, our data highlight that children with higher LNR, and specifically with LNR ≥ 0.2, not only had significantly inferior overall survival but were also more likely to have larger tumors, which are indicative of more aggressive disease. You et al. [14] used Surveillance, Epidemiology, and End Results (SEER) data between 1988 and 2014 and, with classification and regression tree analysis, classified children with Wilms tumors into low and high lymph node density groups using a cutoff of 0.22. The authors were able to demonstrate the association of higher lymph node density with inferior overall survival, but that cohort included children with bilateral disease, metastatic disease, as well as those managed non-operatively over a large time span that included several evolutions in the management of Wilms tumor. Furthermore, our cohort did not demonstrate a survival difference between children with LNR of 0 vs. LNR < 0.2, yet the differences in clinical characteristics and management between these two groups indicate that there is a spectrum in disease biology that warrants further investigation. This finding, along with the increasingly worse overall survival as LNR increases, highlights the spectrum of presentation for children with Wilms tumors and positive lymph nodes, suggesting that LNR may serve as a valuable adjunct in refining risk stratification beyond traditional nodal classification. Our data also emphasized the association of older age at diagnosis with inferior overall survival, which was also recently reported in a SEER database study [31]. Moreover, the fact that increasing tumor size was associated with increased patient mortality in our cohort is expected, since it is a marker of more extensive disease and consistent with previous evidence [13].

Our study has several strengths, including a population-level U.S. cohort and robust statistical analysis. However, our findings need to be considered within the context of certain limitations. The retrospective nature of our study introduces inherent biases, and the AUC value (0.56) suggests that while LNR provides prognostic information, it should be used in conjunction with other established risk factors rather than as a sole determinant of prognosis. It is also worth mentioning the lack of data granularity in NCDB, which did not allow us to evaluate the impact of other important factors on survival in conjunction with LNR, including tumor stage (missing in >80%), treatment strategy, tumor rupture or spillage, tumor histology, as well as loss of heterozygosity at 1p and 16q and gain of chromosome 1q. In addition, the relatively small sample size in the LNR < 0.2 and ≥0.2 groups increase the risk for type II statistical error. Another limitation to consider is the lack of standard guidelines on lymph node sampling, which may lead to difficulties in interpreting the results on LNR. Lastly, while the data included in the NCDB are only collected from institutions accredited by the Commission on Cancer, our findings are still likely generalizable, since these patients require focused, high-level, comprehensive cancer care.

## 5. Conclusions

In conclusion, the findings of this study support the use of LNR as an independent prognostic factor in Wilms tumor, with higher LNR associated with worse overall survival. These results align with prior studies on lymph node density and survival, further emphasizing the importance of adequate lymph node assessment. Future prospective studies are needed to validate these findings and to establish standardized lymph node sampling guidelines that optimize staging accuracy and risk stratification for children with Wilms tumors.

## Figures and Tables

**Table 1:** Demographics and clinical data in children with Wilms tumor by LNR group.

Variable	LNR 0 (n = 1811)	LNR < 0.2 (n = 120)	LNR ≥ 0.2 (n = 275)	Total (n = 2206)	*p*-Value
Age (years)	3.0 (1.0–4.0)	3.0 (2.0–5.0)	3.0 (2.0–5.0)	3.0 (1.0–5.0)	<0.001
Sex					0.57
Male	886 (48.9%)	54 (45.0%)	128 (46.6%)	1068 (48.4%)	
Female	925 (51.1%)	66 (55.0%)	147 (53.4%)	1138 (51.6%)	
Race/ethnicity					0.62
White	1111 (61.4%)	81 (67.5%)	163 (59.3%)	1355 (61.4%)	
Black	346 (19.1%)	17 (14.2%)	54 (19.6%)	417 (18.9%)	
Hispanic	261 (14.4%)	16 (13.3%)	47 (17.1%)	324 (14.7%)	
Other	93 (5.1%)	6 (5.0%)	11 (4.0%)	110 (5.0%)	
Insurance status					0.36
Not insured	32 (1.8%)	4 (3.3%)	3 (1.1%)	39 (1.8%)	
Private insurance	991 (54.7%)	67 (55.8%)	164 (59.6%)	1222 (55.4%)	
Medicaid	711 (39.3%)	41 (34.2%)	97 (35.3%)	849 (38.5%)	
Other	77 (4.3%)	8 (6.7%)	11 (4.0%)	96 (4.4%)	
Charlson–Deyo score					0.83
0	1729 (95.5%)	116 (96.7%)	263 (95.6%)	2108 (95.6%)	
≥1	82 (4.5%)	4 (3.3%)	12 (4.4%)	98 (4.4%)	
Tumor size (cm)	10.2 (7.7–13.0)	12.0 (9.3–14.0)	11.5 (8.9–14.0)	10.5 (8.0–13.0)	<0.001
Number of positive lymph nodes	0.0 (0.0–0.0)	1.0 (1.0–1.0)	2.0 (1.0–4.0)	0.0 (0.0–0.0)	<0.001
Number of lymph nodes examined	4.0 (2.0–8.0)	11.5 (7.5–16.5)	5.0 (3.0–7.0)	5.0 (2.0–8.0)	<0.001
Type of operation					0.28
Partial or subtotal nephrectomy	80 (4.4%)	1 (0.8%)	8 (2.9%)	89 (4.0%)	
Complete/total/simple nephrectomy	332 (18.3%)	19 (15.8%)	47 (17.1%)	398 (18.0%)	
Radical nephrectomy	1338 (73.9%)	93 (77.5%)	209 (76.0%)	1640 (74.3%)	
Nephrectomy and en bloc organ resection	61 (3.4%)	7 (5.8%)	11 (4.0%)	79 (3.6%)	
Surgical margin status					<0.001
Negative	1518 (83.8%)	88 (73.3%)	195 (70.9%)	1801 (81.6%)	
Positive	230 (12.7%)	26 (21.7%)	69 (25.1%)	325 (14.7%)	
Unknown	63 (3.5%)	6 (5.0%)	11 (4.0%)	80 (3.6%)	
Receipt of chemotherapy	1625 (89.7%)	114 (95.0%)	255 (92.7%)	1994 (90.4%)	0.06
Receipt of radiation	710 (39.2%)	116 (96.7%)	254 (92.4%)	1080 (49.0%)	<0.001

**Table 2:** Multivariable Cox regression analysis to assess risk factors of patient mortality.

Variable	Hazard Ratio	95% Confidence Interval	*p*-Value
LNR			
0	Reference	-	-
<0.2	0.76	0.27–2.15	0.61
≥0.2	1.75	1.03–2.97	0.04
Age (years)	1.11	1.05–1.17	<0.001
Sex			
Male	Reference	-	-
Female	1.33	0.88–2.03	0.18
Race/ethnicity			
White	Reference	-	-
Black	1.17	0.71–1.93	0.55
Hispanic	0.96	0.52–1.77	0.90
Other	0.49	0.12–2.02	0.33
Tumor size (cm)	1.03	1.00–1.06	0.03
Chemotherapy	0.92	0.42–2.01	0.84
Radiation	1.43	0.89–2.31	0.14

**Table 3:** Multivariable Cox regression analysis to assess risk factors of patient mortality.

Variable	Hazard Ratio	95% Confidence Interval	*p*-Value
LNR	2.60	1.27–5.32	0.01
Age (years)	1.11	1.05–1.18	<0.001
Sex			
Male	Reference	-	-
Female	1.32	0.87–2.01	0.19
Race/ethnicity			
White	Reference	-	-
Black	1.16	0.70–1.92	0.57
Hispanic	0.97	0.53–1.78	0.91
Other	0.51	0.12–2.09	0.35
Tumor size (cm)	1.03	1.00–1.06	0.03
Chemotherapy	0.95	0.43–2.07	0.89
Radiation	1.38	0.87–2.19	0.17

## Data Availability

The data that support the findings of this study are available from the National Cancer Database. Restrictions apply to the availability of these data, which were used under license for this study. Data from the National Cancer Database Patient User Data Files are available upon request at https://ncdbapp.facs.org/puf/.

## Supplementary Materials

The following supporting information can be downloaded at: https://www.mdpi.com/article/10.3390/cancers17193276/s1, Supplemental File S1: R code function to determine and evaluate optimal cut-points.
