# Supplementary material for: The Impact of Lymph Node Ratio for Children with Wilms Tumors: A National Cancer Database Analysis
Source: Cancers (Basel). 2025 Oct 9;17(19):3276. doi: 10.3390/cancers17193276 (PMC12523910; doi:10.3390/cancers17193276)
Supplement: Supplementary file 1 [file cancers-17-03276-s001.zip › cancers-3894459-supplementary.pdf]

Supplemental File S1. R code function to determine and evaluate optimal cut-points.

```
cutpointr(data, x = LNR, # data, variable used for classification
 class = Mortality, # variable indicating class membership
 method = maximize_metric, # function used to determine cutpoint = maximize the
metric
 metric = sum_sens_spec, # metric to compute = sensitivity + specificity
 tol_metric = 0.01, # cutpoints returned are within 0.01 of maximum achievable metric
 break_ties = c) # if multiple cutpoints are found, return all cutpoints
```
